# Anticholinergic Burden Does Not Influence Delirium Subtype or the Delirium–Mortality Association in Hospitalized Older Adults: Results from a Prospective Cohort Study

**DOI:** 10.1007/s40266-020-00827-1

**Published:** 2021-01-08

**Authors:** Mark James Rawle, Laura McCue, Elizabeth L. Sampson, Daniel Davis, Victoria Vickerstaff

**Affiliations:** 1 MRC Unit for Lifelong Health and Ageing at UCL, London, UK; 2 Whipps Cross University Hospital, London, UK; 3 Marie Curie Palliative Care Research Department, UCL, London, UK; 4 Barnet, Enfield and Haringey Mental Health Trust, London, UK; 5 Research Department of Primary Care and Population Health, UCL, London, UK

## Abstract

**Background:**

Anticholinergic burden (ACB) is associated with an increased risk of delirium in the older population outside of the acute hospital setting. In acute settings, delirium is associated with increased mortality, and this association is greater with full syndromal delirium (FSD) than with subsyndromal delirium (SSD). Little is known about the impact of ACB on delirium prevalence or subtype in hospitalized older adults or the impact on mortality in this population.

**Objectives:**

Our objectives were to determine whether ACB moderates associations between the subtype of delirium experienced by hospitalized older adults and to explore factors (including ACB) that might moderate consequent associations between delirium and mortality in hospital inpatients.

**Methods:**

We conducted a retrospective analysis of a cohort of 784 older adults with unplanned admission to a North London acute medical unit between June and December 2007. Univariate regression analyses were performed to explore associations between ACB, as represented by the Anticholinergic Burden Scale (ACBS), delirium subtype (FSD vs. SSD), and mortality.

**Results:**

The mean age of the sample was 83 ± standard deviation (SD) 7.4 years, and the majority of patients were female (59%), lived in their own homes (71%), were without dementia (75%), and died between hospital admission and the end of the 2-year follow-up period (59%). Mean length of admission was 13.2 ± 14.4 days. Prescription data revealed an ACBS score of 1 in 26% of the cohort, of 2 in 12%, and of ≥ 3 in 16%. The mean total ACBS score for the cohort was 1.1 ± 1.4 (range 0–9). Patients with high ACB on admission were more likely to have severe dementia, to have multiple comorbidities, and to live in residential care. Higher ACB was not associated with delirium of either subtype in hospitalized older adults. Delirium itself was associated with increased mortality, and greater associations were seen in FSD (hazard ratio [HR] 2.27; 95% confidence interval [CI] 1.70–3.01) than in SSD (HR 1.58; 95% CI 1.2–2.09); however, ACB had no impact on this relationship.

**Conclusions:**

ACB was not found to be associated with increased delirium of either subtype or to have a demonstrable impact on mortality in delirium. Prior suggestions of links between ACB and mortality in similar populations may be mediated by higher levels of functional dependence, greater levels of residential home residence, or an increased prevalence of dementia in this population.

## Key Points

**Table:** 

Anticholinergic medication use is not associated with delirium of any subtype.
In older adults admitted to hospital, delirium is associated with greater mortality.
Of these individuals, those who are also prescribed anticholinergic medication are no more or less likely to die.

## Background

Delirium is a complex neuropsychiatric syndrome characterized by acute onset of fluctuating inattention and cognitive deficits [1]. These deficits include disorganized thinking, perceptual disturbances, altered levels of consciousness, and changes in social and physical behaviors [2]. Delirium can be categorized based on variability in motor and arousal presentations (hyper- and hypoactive subtypes) or by subtypes such as full syndromal delirium (FSD) and subsyndromal delirium (SSD). Acute onset and inattention, in addition to disorganized thinking or altered consciousness, indicate FSD. The presence of one or more, but not all of these symptoms, indicates SSD [3].

Delirium is common in hospitalized older adults, particularly those admitted on an unplanned or emergency basis (60–87%) [4]. Delirium has consistently been shown to increase mortality risk, doubling mortality during and beyond the initial period of hospitalization [5–7]. This could be because delirium indicates underlying disease severity, or it could be related to other issues independently associated with increased mortality in older people, such as dementia [8], frailty [6], and infection [9]. Factors that increase delirium risk include preexisting cognitive impairment or dementia, physical and mental comorbidities, functional dependence, infection, and polypharmacy [4, 10–14]. For polypharmacy, medications with anticholinergic properties are particularly relevant.

Anticholinergic drugs are prescribed to 20–50% of older people to treat a range of medical illnesses affecting autonomic smooth muscle, neuromuscular, or central nervous system function. These include urinary incontinence [15], Parkinson’s disease, respiratory disorders [16], and depression [17]. Age-related physiological changes, including increased blood–brain barrier permeability, and reduced renal and hepatic clearance, result in the use of these medications being associated with a cumulative anticholinergic burden (ACB) [18]. ACB is associated with increased risk of incident delirium in the older population [4, 19–23], with estimates for experiencing delirium increasing incrementally by up to 40% for each additional anticholinergic medication taken [20]. ACB is also associated with impaired physical function [24] and mortality [25].

The relationship between ACB and a higher lifetime risk of incident delirium has been previously noted across different countries [4, 19, 21, 26], clinical diagnoses [22, 27], and residential settings [21, 28], yet existing studies have found no evidence for a relationship between delirium during hospital admission and ACB [29–32]. In addition, studies exploring the relationship between delirium and mortality in hospitals neglect ACB as a potential mediator [33–36], despite its known association with mortality. With the exception of one study [37], existing studies have explored delirium prevalence as a binary outcome. Little research has examined the influence of ACB upon different subtypes of delirium. We had two aims: (1) to determine whether ACB was associated with the subtype of delirium (FSD vs. SSD) experienced by hospitalized older adults and (2) to explore whether ACB moderated consequent associations between delirium and mortality in hospital inpatients.

## Methods

### Study Design, Setting, and Population

This was a retrospective analysis of prospectively collected cohort data. Data were analyzed from a cohort of 784 older adults with an emergency unplanned admission (via the emergency department or clinic) to a North London acute medical unit between June and December 2007. Participants were aged ≥ 70 years and were admitted for at least 48 h. We excluded participants with no available information on medications prescribed on admission. Within 72 h of admission, participants underwent clinical assessments by trained clinicians (old age psychiatrists) to gather relevant baseline data. Prior to assessment, verbal consent was gained from the participant or, where the participant lacked capacity, their carer. Ethical approval was provided by the Royal Free Hospital NHS Trust Ethics Committee (06/Q0501/31).

### Ascertainment of Delirium

Delirium was evaluated using the Confusion Assessment Method short-form (s-CAM) [38], which assesses common features of delirium, specifically acute onset, inattention, disorganized thinking, and altered level of consciousness. The s-CAM has high sensitivity (95%) and specificity (89%) for delirium when delivered by trained researchers [39]. The s-CAM can also be used to effectively diagnose delirium in people with dementia [40]. Based on the number of features present within this diagnostic assessment, a subtype of either FSD (three or more features) or SSD (two features) was assigned, a method previously found to have high accuracy for subtype differentiation [3].

### Ascertainment of Anticholinergic Burden on Admission

Total ACB was calculated and categorized (0, 1, 2, ≥ 3) using the Anticholinergic Cognitive Burden Scale (ACBS), which scores individual medications based on their likelihood of a clinically relevant anticholinergic effect from 0 (no effect) to 3 (suggestive of anticholinergic effect causing delirium) [41]. While other measures of anticholinergic activity exist, the ACBS was chosen because of its frequency of use in existing literature on associations with delirium and because anticholinergic medications within the scale are graded on their potential to cause cognitive rather than peripheral effects [42].

### Ascertainment of Mortality

Mortality data were linked to the UK Office for National Statistics, which allowed for automatic notification of a participant’s death for up to 2 years following the beginning of the study. We chose 2 years a priori as delirium has strong predictive value for 1-year mortality [43] and limited evidence for associations with mortality up to 2 years post discharge [44]. Survival time was from the date of hospital admission to the date of death or until censoring on 9 July 2009.

### Covariates

Demographic data associated with both delirium and mortality were collected from participants’ hospital records, including age, sex, place of residence, ethnicity, marital status, smoking status, and level of education. Data for other medical conditions that might impact both delirium and mortality were collected. These included severity of acute illness measured using the Acute Physiology and Chronic Health Evaluation (APACHE-II) [45], patient burden of comorbidities measured using the Charlson Comorbidity Index (CCI) [46], pressure ulcer risk measured using the Waterlow Scale [47], total medication count and the Functional Assessment Staging Tool (FAST) [48] to examine stage of dementia prior to admission to hospital. These measures were treated as continuous variables for statistical analysis, bar the FAST, which used a score of ≥ 6a to represent the highest degrees of functional impairment. Researchers ascertained dementia status using a structured clinical assessment based on operationalized *Diagnostic and Statistical Manual of Mental Disorders, 4th edition* (DSM-IV) criteria [49] incorporating the Mini-Mental State Examination [50], a review of medical notes, and a discussion with the participant and their carers. Length of hospital admission was assessed via hospital records.

### Statistical Analyses

Demographic and clinical characteristics of the cohort were described using measures of central tendency and variability to explore differences per ACBS category. Analysis of variance (ANOVA), Kruskal–Wallis, and Pearson’s chi-squared tests were used, as appropriate, to test for relationships between continuous and categorical outcome variables and ACBS category. Histograms were plotted to assess normality so we could select the appropriate test. We used univariable logistic regressions to test associations between ACB and delirium subtype. A series of multinomial logistic regressions were estimated to assess independent associations between all covariates (detailed in Sect. 2.5) and both delirium subtypes. Cox proportional hazard regressions were used for all covariates and mortality outcomes. ANOVA, Kruskal–Wallis, and Pearson’s chi-squared tests were used, as appropriate, to test for relationships between continuous and categorical outcome variables and between delirium status and mortality status. All analyses were performed using STATA version 15 [51] on a complete-case basis, including only participants with full data on delirium status and medication.

## Results

### Participant Eligibility

**Fig. 1:**
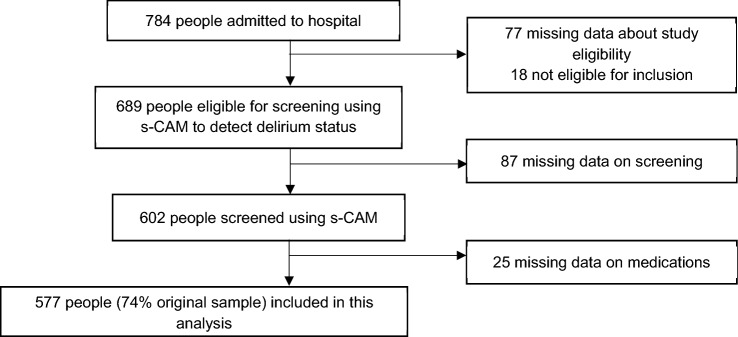
Study flowchart showing participant eligibility and exclusion process. *s-CAM* Confusion Assessment Method short-form

During the study period, 784 potential participants were admitted to hospital and met inclusion criteria. Of these, 577 participants had full admission data, including s-CAM screening and medication data (74% of original sample), and were included for final analysis (Fig. 1).

### Cohort and Clinical Characteristics

Demographic and clinical characteristics of the cohort categorized by ACBS score are provided in Table 1. The mean age of the sample was 83 ± standard deviation (SD) 7.4 years, and the majority were female (59%), lived in their own homes (71%), were not diagnosed with dementia (75%), and died between hospital admission and the end of the 2-year follow-up period (59%). Median length of admission was 8 days (interquartile range 4–16).

**Table 1:** Cohort and clinical characteristics by Anticholinergic Burden Scale score

Variables	Total	Anticholinergic Burden Scale Score				*p* value
		0	1	2	3+	
*n* (%)	577 (100)	264 (46)	151 (26)	71 (12)	91 (16)	
Age (years)	83.2 ± 7.4	83.7 ± 7.5	83.1 ± 7.4	81.3 ± 6.8	83.7 ± 7.3	0.083
Sex						0.073
Male	237 (41)	117 (44)	55 (36)	35 (49)	30 (33)	
Female	340 (59)	147 (56)	96 (64)	36 (51)	61 (67)	
Residence type (*n* = 576)						0.002
House	408 (71)	190 (72)	106 (70)	60 (85)	52 (57)	
Residential care	168 (29)	73 (28)	45 (30)	11 (15)	39 (43)	
Ethnicity						0.021
White	518 (90)	239 (91)	128 (85)	63 (89)	88 (97)	
Other	59 (10)	25 (9)	23 (15)	8 (11)	3 (3)	
Marital status (*n* = 571)						0.183
Single	84 (15)	43 (16)	20 (13)	5 (7)	16 (18)	
Other	487 (85)	218 (84)	129 (87)	66 (93)	74 (82)	
Smoking status (*n* = 569)						0.084
Never	263 (46)	116 (44)	73 (50)	26 (37)	48 (53)	
Ex	256 (45)	115 (44)	62 (42)	42 (59)	37 (41)	
Current	50 (9)	30 (11)	12 (8)	3 (4)	5 (6)	
Delirium diagnosis						0.484
FSD	77 (13)	34 (13)	21 (14)	7 (10)	15 (16)	
SSD	98 (17)	44 (17)	22 (15)	11 (15)	21 (23)	
No delirium	402 (70)	186 (70)	108 (72)	53 (75)	55 (60)	
Mortality						0.402
Deceased	339 (59)	161 (61)	80 (53)	44 (62)	54 (59)	
Not deceased	238 (41)	103 (39)	71 (47)	27 (38)	37 (41)	
Dementia diagnosis						0.001
Yes	143 (25)	68 (26)	32 (21)	8 (11)	35 (38)	
No	434 (75)	196 (74)	119 (79)	63 (89)	56 (62)	
MMSE score	19.2 ± 10.2; 23 (14–27)	19.3 ± 10.3; 23 (14–27)	19.6 ± 10.2; 24 (15–27)	21.3 ± 8.9; 24 (19–27)	16.8 ± 10.5; 21 (6–25)	0.038
FAST stage (*n* = 556)						0.006
1	243 (44)	114 (45)	72 (50)	33 (47)	24 (27)	
2–5	167 (30)	72 (28)	41 (28)	25 (36)	29 (33)	
≥ 6a	146 (26)	68 (27)	31 (22)	12 (17)	35 (40)	
Waterlow score (*n* = 570)	13.1 ± 6.4	13.3 ± 6.5	12.3 ± 6.2	12.1 ± 5.5	14.5 ± 6.7	0.03
APACHE-II score (*n* = 569)	12.1 ± 3.7	12.1 ± 3.5	12.1 ± 4.0	12.1 ± 4.2	12.1 ± 3.1	0.996
Days of admission (*n* = 576)	13.2 ± 14.4; 8 (4–16)	13.2 ± 14.4; 8 (4–16)	12.8 ± 14.1; 7 (4–15)	14.4 ± 16.8; 9 (4–14)	9 ± 16; 9 (4–20)	0.815
Medications on admission (*n* = 577)	5.9 ± 3.0; 5 (4–8)	4.6 ± 2.8; 4 (2–6)	6.1 ± 2.6; 6 (4–8)	7.7 ± 2.7; 8 (6–9)	7.9 ± 2.7; 8 (5–10)	< 0.001

Data are presented as mean ± standard deviation, *N* (%), or median (interquartile range) unless otherwise indicated. Total *n* = 577 unless otherwise stated. Chi-squared test, Fisher’s exact test, ANOVAs or the Kruskal–Wallis test were performed as appropriate

FAST stage: 1 = no functional impairment; 2–5 = subjective/objective functional deficit, difficulties with activities of daily living; ≥ 6a = Help required dressing, toileting, personal hygiene, limited vocabulary, can no longer walk, sit up, or hold head up

*ANOVA* analysis of variance, *APACHE-II* Acute Physiology and Chronic Health Evaluation II, *FAST* Functional Assessment Staging Tool, *FSD* full syndromal delirium, *MMSE* Mini-Mental State Examination, *SSD* subsyndromal delirium

Nearly half of the cohort were not prescribed any medications with anticholinergic properties (46%). Prescription data revealed an ACBS score of 1 in 26%, of 2 in 12%, and of ≥ 3 in 16%. The mean ± SD total ACBS score for the cohort was 1.1 ± 1.4 (range 0–9), and the median was 1 (interquartile range 0–2). Participants with an ACBS score of ≥ 3 were more likely to be female, single, and living in residential or care homes. Participants with higher ACBS scores were more likely to have more severe dementia and multiple comorbidities.

Cohort characteristics stratified by (1) delirium subtype and (2) mortality are provided in Table 2. FSD was present in 13% (*n* = 77) and SSD in 17% (*n* = 98). Compared with those without delirium, participants with FSD were more likely to be older and at greater risk of pressure sores and to have more severe acute illness, more comorbidities, and a longer hospital stay. Individuals with FSD were also more likely to be living in residential or nursing care and have a dementia diagnosis. These patterns were also seen in those with SSD but to a lesser degree. When stratified by mortality, the same patterns were seen in the 59% (*n* = 339) of the cohort who had died within the 2-year follow-up period.

**Table 2:** Cohort and clinical characteristics by delirium subtype and mortality status

Variables	Delirium status				Mortality		
	FSD	SSD	No delirium	*p* value	Deceased	Not deceased	*p* value
*n* (%)	77 (13)	98 (17)	402 (70)		339 (59)	238 (41)	
Age (years)	86.9 ± 7.0	83.7 ± 7.4	82.4 ± 7.3	< 0.001	84.1 ± 7.4	82.0 ± 7.2	< 0.001
Female	48 (62)	70 (71)	222 (55)	0.011	200 (59)	140 (59)	0.967
Residence type (*n* = 576)				< 0.001			< 0.001
House	33 (43)	59 (60)	316 (79)		213 (63)	195 (82)	
Residential home	13 (17)	11 (11)	18 (4)		33 (10)	9 (4)	
Nursing home	25 (32)	26 (27)	31 (8)		65 (19)	17 (7)	
Sheltered	5 (6)	2 (2)	37 (9)		27 (8)	17 (7)	
ACBS score	1.2 ± 1.6	1.2 ± 1.4	1.1 ± 1.4	–	1.1 ± 1.4	1.1 ± 1.4	–
Median (IQR)	1 (0–2)	1 (0–2)	1 (0–2)	0.684	1 (0–2)	1 (0–2)	0.605
Dementia diagnosis	46 (60)	38 (39)	59 (15)	< 0.001	99 (29)	44 (18)	0.003
FAST stage (*n* = 556)				< 0.001			< 0.001
1	2 (3)	15 (16)	226 (57)		118 (36)	125 (54)	
2–5	12 (17)	32 (34)	123 (31)		98 (30)	69 (30)	
≥ 6a	55 (80)	46 (50)	45 (11)		109 (34)	37 (16)	
Waterlow score (*n* = 570)	17.4 ± 7.1	16.6 ± 7.3	11.4 ± 5.2	< 0.001	14.4 ± 6.6	11.1 ± 5.4	< 0.001
CCI score	3.0 ± 1.3	3.3 ± 2.5	2.7 ± 2.1	0.052	3.3 ± 2.2	2.2 ± 1.9	< 0.001
APACHE-II score (*n* = 569)	14.3 ± 4.4	13.5 ± 4.1	11.3 ± 3.1	< 0.001	12.7 ± 4.0	11.3 ± 3.0	< 0.001
Days of admission (*n* = 576)	18.5 ± 16.8	14.8 ± 13.0	12.4 ± 14.9	–	15.4 ± 15.5	11.0 ± 13.9	–
Median (IQR)	7 (4–14)	11 (5–21)	13 (7–25)	< 0.001	6 (3–12)	10 (5–19)	< 0.001

Data are presented as mean ± standard deviation, N (%) unless otherwise indicated. Total n = 577 unless otherwise stated. Pearson chi-squared, ANOVA, and Kruskal–Wallis tests used where appropriate

*ACBS* Anticholinergic Cognitive Burden Scale, *APACHE-II* Acute Physiology and Chronic Health Evaluation II, *CCI* Charlson Comorbidity Index, *FAST* Functional Assessment Staging Tool, *FSD* full syndromal delirium, *IQR* interquartile range, *SSD* subsyndromal delirium

### Associations Between Anticholinergic Burden and Delirium Subtype

The presence of any delirium was not associated with higher ACBS scores in hospitalized inpatients. The presence of delirium was instead strongly associated with the highest degrees of functional impairment, represented by a FAST score of ≥ 6a (odds ratio [OR] SSD 7.59; 95% confidence interval [CI] 4.55–12.7 vs. FSD 30.5; 95% CI 15.7–59.2), nursing home residency (OR SSD 2.35; 95% CI 1.52–3.88 vs. FSD 4.79; 95% CI 2.87–7.99), the presence of dementia (OR SSD 3.68; 95% CI 2.25–6.02 vs. FSD 8.63; 95% CI 5.06–14.7), and increasing age (OR per additional year of age, SSD 1.02; 95% CI 0.99–1.06 vs. FSD 1.09; 95% CI 1.05–1.13). Female sex was also associated with delirium but with a higher prevalence of SSD than FSD (OR SSD 2.02; 95% CI 1.25–3.28 vs. FSD 1.34; 95% CI 0.81–2.22). More severe acute illness, represented by increased APACHE-II scores and increased Waterlow scores, was also associated with higher rates of delirium, with little difference in delirium subtype. Higher levels of comorbidity, as represented by CCI score, were weakly associated with delirium. Univariable analyses are provided in Table 3.

**Table 3:** Univariable regression models to explore the effect of study variables on delirium and mortality

Variable	Delirium (no delirium is base outcome)			Mortality^a^	*p* value
	SSD^b^	FSD^b^	*p* value		
Presence of delirium (ref. = none)	–				< 0.001
SSD	–			1.58 (1.20–2.09)	
FSD	–			2.27 (1.70–3.01)	
Age (per 1 year increase)	1.02 (0.99–1.06)	1.09 (1.05–1.13)	< 0.001	1.03 (1.02–1.05)	< 0.001
Sex (Ref. = male)			0.0123		0.794
Female	2.02 (1.25–3.28)	1.34 (0.81–2.22)		1.03 (0.83–1.28)	
Residential type (ref.= living at home) (*n* = 576)			< 0.001		< 0.001
Residential care	2.32 (1.52–3.88)	4.79 (2.87–7.99)		1.83 (1.27–2.65)	
Dementia (ref. = no dementia)	3.68 (2.25–6.02)	8.63 (5.06–14.7)	< 0.001	1.45 (1.15–1.84)	0.002
FAST stage (ref. = 1–5) (*n* = 556)			< 0.001		
≥ 6a	7.59 (4.55–12.7)	30.5 (15.7–59.2)	< 0.001	1.80 (1.43–2.27)	< 0.001
Waterlow score (*n* = 570)	1.15 (1.11–1.19)	1.17 (1.12–1.22)	< 0.001	1.06 (1.05–1.08)	< 0.001
CCI score	1.12 (1.02–1.24)	1.07 (0.95–1.19)	0.054	1.17 (1.12–1.23)	< 0.001
APACHE-II score (*n* = 569)	1.19 (1.12–1.27)	1.24 (1.16–1.32)	< 0.001	1.08 (1.05–1.11)	< 0.001
ACBS score (ref. = 0)			0.493	0.884 (0.68–1.16)	0.744
1	0.86 (0.49–1.51)	1.06 (0.59–1.93)			
2	0.88 (0.42–1.82)	0.72 (0.30–1.72)		1.05 (0.75–1.47)	
≥ 3	1.61 (0.89–2.94)	1.49 (0.76–2.94)		1.03 (0.75–1.40)	
Number of medications on admission (per 1 medication increase)	1.04 (0.97–1.12)	1.02 (0.94–1.11)	0.546	1.03 (1.00–1.07)	0.074

Total *n* = 577 unless otherwise stated. Statistics obtained through a series of logistic or Cox regressions. *p* values indicate significance of association between outcome and covariate

*ACBS* Anticholinergic Cognitive Burden Scale, *APACHE-II* Acute Physiology and Chronic Health Evaluation II, *CCI* Charlson Comorbidity Index, *FAST* Functional Assessment Staging Tool, *FSD* full syndromal delirium, *Ref* reference group, *SSD* subsyndromal delirium

^a^Hazard ratio (95% confidence interval)

^b^Odds ratio (95% confidence interval)

### The Effect of Anticholinergic Burden on Associations Between Delirium Subtype and Mortality

Higher ACBS scores were not associated with an increase in mortality in unadjusted analyses. Higher mortality was associated with delirium, with increased levels in FSD (hazard ratio [HR] 2.27; 95% CI 1.70–3.01) versus SSD (HR 1.58; 95% CI 1.20–2.09) as previously seen in this sample [5–7]. Increased mortality was also associated with nursing home residency (HR 2.13; 95% CI 1.61–2.82), dementia (HR 1.45; 95% CI 1.15–1.84), and increasing age (HR per additional year of age 1.03; 95% CI 1.02–1.05). Again, worse CCI, APACHE-II, Waterlow, and FAST scores were associated with increased levels of mortality. Univariable analyses are provided in Table 3. Delirium remained strongly associated with mortality after adjusting for residential status, Waterlow score, CCI, and the presence of dementia. Within all delirium subtypes, controlling for ACBS score did not notably affect the mortality hazard, suggesting that anticholinergic burden is not a mediator or confounder of the previously established relationship between delirium and mortality.

## Discussion

Our study explored the effects of medications with anticholinergic properties upon delirium while also considering subsyndromal presentations and is the first to investigate the effect of these medications in the relationship between delirium subtypes and mortality. In a large sample of older adults admitted to an acute hospital, ACBS score was not associated with any delirium subtype in bivariate analyses. Delirium and mortality were strongly associated, with a dose–response relationship between delirium subtype and mortality risk; patients with FSD were over three times more likely to die than those with SSD or no delirium. Yet, relationships between delirium and mortality were not attenuated by ACBS score for any of the delirium subtypes. This suggests that, among hospitalized older adults, ACB may not affect delirium subtype or the relationship between delirium and mortality.

ACBS score was not associated with the presence of any type of delirium in our study, and this finding supports and extends the findings of other smaller studies that have shown no effect of anticholinergic medications upon delirium in hospitalized older adults [20, 29–33]. Unlike our study, some studies have found evidence for an association between higher ACB and increased risk of delirium in this population [4, 19, 23, 27, 37, 52]. While these studies accounted for factors such as sensory impairment and prior antipsychotic use, they did not account for severity of acute illness, dementia, and the risk of pressure sores. The sample size of these studies was also smaller, and different anticholinergic drug scales with poor agreement [53] are used across the literature, which may have contributed to these conflicting results. The delirium–mortality findings are in line with those of existing literature [5–7]. ACB did not mediate or confound the relationship between delirium and mortality in our study. In agreement with existing literature, numerous other demographic and clinical characteristics were significantly and independently related to delirium, such as severity of acute illness and comorbidity [10, 13, 54]. It is plausible that the delirium–mortality relationship is driven by these factors over ACB.

Despite the notable strengths of a large sample size and robust measures of delirium, ACB, and confounders, our study is not without limitations. The ACBS is the most frequently used validated expert-based ACB scale [55]. However, it is used here as a measure of medication use on admission to hospital and therefore does not take into account medication dosage, compliance, length of usage, or any changes in drugs during/post hospitalization. Our measure of delirium was as point prevalence via comprehensive assessment methods undertaken by trained and experienced old age psychiatrists. Verification of information provided by the participant with family members, staff, and medical records limited recall bias from older participants experiencing delirium or memory difficulties. However, these assessments were performed upon hospital admission, and delirium symptoms may peak during the 2nd week of hospitalization [4], so incident delirium that occurred after the recruitment period may have been missed. Yet the prevalence of delirium in this study (30%) was similar to that found in other studies in similar settings and populations [20, 23, 26]. Despite a relatively comprehensive assessment of potential confounders of the relationships between ACB, delirium, and mortality, residual confounding remains possible. Frailty [6], social or environmental factors such as isolation [56], the use of physical restraints [57], alcohol [58], and pain [59] may play a role in the relationship between studied variables. Our data came from a predominantly White British population from a London hospital; however, this hospital serves 1.2 million people, and our sample size is notably larger than many studies in the field.

Our findings suggest that heavy ACB does not contribute to an increased risk of delirium or directly influence the delirium–mortality association among older acute hospital inpatients. Our findings also raise doubts regarding the notion that a higher ACB may differ in terms of SSD and FSD in older hospitalized populations in a dose–response fashion. This lack of association may have been a consequence of methodological limitations in the recording of ACB. It may still be that individual medications with a strongly anticholinergic effect, such as benzodiazepines or older antihistamines, mediate relationships between ACB, delirium, and mortality. Individuals with an ACBS score of 3 who are on three medications with a speculated anticholinergic effect (ACBS score 1, i.e., furosemide, codeine, and omeprazole) may not experience the same anticholinergic effect as those on a single confirmed anticholinergic (ACBS score 3, i.e., oxybutynin). Limited evidence exists that alternative measures of ACB (such as the Anticholinergic Risk Scale, which grades drugs on total anticholinergic effect rather than cognitive effect) might be more consistently associated with delirium in hospital populations [42]; however these data are still emerging, and the ACBS remains the most frequently used scale in existing literature. In addition, anticholinergic therapy may still have an impact on mortality but via a less direct method. Any mortality increase may be mediated in our study by higher levels of functional dependence, residential home residence, or an increased prevalence of dementia, all of which have been previously linked independently to both high anticholinergic exposure and mortality.

## Conclusions

In our study, ACB was not associated with increased delirium of either subtype, nor did it have demonstrable impact on mortality in delirium. Prior suggestions of links between ACB and mortality in similar populations may be mediated by higher levels of functional dependence, residential home residence, or dementia in these populations.

Future studies might focus on including more detailed assessments of drug composition within their measure of ACBS or explore these relationships in a population determined to be at higher risk from anticholinergic effects (i.e., hospital inpatients with dementia). Increased awareness of the pathways in which ACB may contribute to the occurrence of delirium and consequent adverse outcomes will allow for improvements in quality of individualized treatment.

## Declarations

### Availability of data and material

The datasets generated and analyzed during the current study are available from the corresponding author on reasonable request.

### Code availability

The code generated and analyzed during the current study are available from the corresponding author on reasonable request.

## References

[1] Fong TG, Tulebaev SR, Inouye SK (2009). Delirium in elderly adults: diagnosis, prevention and treatment. Nat Rev Neurol..

[2] Inouye SK, van Dyck CH, Alessi CA, Balkin S, Siegal AP, Horwitz RI (1990). Clarifying confusion: the confusion assessment method. A new method for detection of delirium. Ann Intern Med..

[3] Meagher D, O'Regan N, Ryan D, Connolly W, Boland E, O'Caoimhe R (2014). Frequency of delirium and subsyndromal delirium in an adult acute hospital population. Br J Psychiatry..

[4] Naja M, Zmudka J, Hannat S, Liabeuf S, Serot JM, Jouanny P (2016). In geriatric patients, delirium symptoms are related to the anticholinergic burden. Geriatr Gerontol Int..

[5] Witlox J, Eurelings LS, de Jonghe JF, Kalisvaart KJ, Eikelenboom P, van Gool WA (2010). Delirium in elderly patients and the risk of postdischarge mortality, institutionalization, and dementia: a meta-analysis. JAMA.

[6] Dani M, Owen LH, Jackson TA, Rockwood K, Sampson EL, Davis D (2018). Delirium, frailty, and mortality: interactions in a prospective study of hospitalized older people. J Gerontol A Biol Sci Med Sci..

[7] Diwell RA, Davis DH, Vickerstaff V, Sampson EL (2018). Key components of the delirium syndrome and mortality: greater impact of acute change and disorganised thinking in a prospective cohort study. BMC Geriatr..

[8] Fong TG, Davis D, Growdon ME, Albuquerque A, Inouye SK (2015). The interface between delirium and dementia in elderly adults. Lancet Neurol..

[9] Kuswardhani RAT, Sugi YS (2017). Factors related to the severity of delirium in the elderly patients with infection. Gerontol Geriatr Med..

[10] Uguz F, Kayrak M, Cicek E, Kayhan F, Ari H, Altunbas G (2010). Delirium following acute myocardial infarction: incidence, clinical profiles, and predictors. Perspect Psychiatr Care..

[11] O'Sullivan R, Inouye SK, Meagher D (2014). Delirium and depression: inter-relationship and clinical overlap in elderly people. Lancet Psychiatry..

[12] Hein C, Forgues A, Piau A, Sommet A, Vellas B, Nourhashemi F (2014). Impact of polypharmacy on occurrence of delirium in elderly emergency patients. J Am Med Dir Assoc..

[13] Ahmed S, Leurent B, Sampson EL (2014). Risk factors for incident delirium among older people in acute hospital medical units: a systematic review and meta-analysis. Age Ageing.

[14] Laurila JV, Laakkonen ML, Tilvis RS, Pitkala KH (2008). Predisposing and precipitating factors for delirium in a frail geriatric population. J Psychosom Res..

[15] Campbell N, Boustani M, Limbil T, Ott C, Fox C, Maidment I (2009). The cognitive impact of anticholinergics: a clinical review. Clin Interv Aging..

[16] Gerretsen P, Pollock BG (2011). Drugs with anticholinergic properties: a current perspective on use and safety. Expert Opin Drug Saf..

[17] Kouladjian O'Donnell L, Gnjidic D, Nahas R, Bell JS, Hilmer SN (2017). Anticholinergic burden: considerations for older adults. J Pharm Pract Res..

[18] Collamati A, Martone AM, Poscia A, Brandi V, Celi M, Marzetti E (2016). Anticholinergic drugs and negative outcomes in the older population: from biological plausibility to clinical evidence. Aging Clin Exp Res..

[19] Best O, Gnjidic D, Hilmer SN, Naganathan V, McLachlan AJ (2013). Investigating polypharmacy and drug burden index in hospitalised older people. Intern Med J..

[20] Egberts A, van der Craats ST, van Wijk MD, Alkilabe S, van den Bemt PM, Mattace-Raso FU (2017). Anticholinergic drug exposure is associated with delirium and postdischarge institutionalization in acutely ill hospitalized older patients. Pharmacol Res Perspect..

[21] Landi F, Dell'Aquila G, Collamati A, Martone AM, Zuliani G, Gasperini B (2014). Anticholinergic drug use and negative outcomes among the frail elderly population living in a nursing home. J Am Med Dir Assoc..

[22] Crispo JA, Willis AW, Thibault DP, Fortin Y, Hays HD, McNair DS (2016). Associations between anticholinergic burden and adverse health outcomes in Parkinson disease. PLoS ONE.

[23] Rojo-Sanchis AM, Velez-Diaz-Pallares M, Munoz Garcia M, Delgado Silveira E, Bermejo Vicedo T, Cruz Jentoft A (2016). Anticholinergic burden and delirium in elderly patients during acute hospital admission. Rev Esp Geriatr Gerontol.

[24] Lampela P, Lavikainen P, Garcia-Horsman JA, Bell JS, Huupponen R, Hartikainen S (2013). Anticholinergic drug use, serum anticholinergic activity, and adverse drug events among older people: a population-based study. Drugs Aging.

[25] Mangoni AA, van Munster BC, Woodman RJ, de Rooij SE (2013). Measures of anticholinergic drug exposure, serum anticholinergic activity, and all-cause postdischarge mortality in older hospitalized patients with hip fractures. Am J Geriatr Psychiatry..

[26] Zimmerman KM, Salow M, Skarf LM, Kostas T, Paquin A, Simone MJ (2014). Increasing anticholinergic burden and delirium in palliative care inpatients. Palliat Med..

[27] Caeiro L, Ferro JM, Claro MI, Coelho J, Albuquerque R, Figueira ML (2004). Delirium in acute stroke: a preliminary study of the role of anticholinergic medications. Eur J Neurol..

[28] Huang K-H, Chan Y-F, Shih H-C, Lee C-Y. Relationship between potentially inappropriate anticholinergic drugs (PIADs) and adverse outcomes among elderly patients in Taiwan. J Food Drug Anal. 2012;20(4).

[29] Moorey HC, Zaidman S, Jackson TA (2016). Delirium is not associated with anticholinergic burden or polypharmacy in older patients on admission to an acute hospital: an observational case control study. BMC Geriatr..

[30] Gaudreau JD, Gagnon P, Harel F, Roy MA, Tremblay A (2005). Psychoactive medications and risk of delirium in hospitalized cancer patients. J Clin Oncol..

[31] Campbell N, Perkins A, Hui S, Khan B, Boustani M (2011). Association between prescribing of anticholinergic medications and incident delirium: a cohort study. J Am Geriatr Soc.

[32] Pasina L, Colzani L, Cortesi L, Tettamanti M, Zambon A, Nobili A (2019). Relation between delirium and anticholinergic drug burden in a cohort of hospitalized older patients: an observational study. Drugs Aging.

[33] Luukkanen M, Uusvaara J, Laurila J, Strandberg T, Raivio M, Tilvis R (2011). Anticholinergic drugs and their effects on delirium and mortality in the elderly. Dement Geriatr Cogn Disord Extra..

[34] Klouwenberg PMK, Zaal IJ, Spitoni C, Ong DS, Van Der Kooi AW, Bonten MJ (2014). The attributable mortality of delirium in critically ill patients: prospective cohort study. BMJ.

[35] Eeles EM, Hubbard RE, White SV, O’Mahony MS, Savva GM, Bayer AJ (2010). Hospital use, institutionalisation and mortality associated with delirium. Age Ageing.

[36] Mulligan O, Muresan L, Murray O, Adamis D, McCarthy G (2015). Mortality at one year post delirium in general medical inpatients. Eur Psychiatry..

[37] Han L, McCusker J, Cole M, Abrahamowicz M, Primeau F, Elie M (2001). Use of medications with anticholinergic effect predicts clinical severity of delirium symptoms in older medical inpatients. Arch Intern Med.

[38] Inouye SK, Director ABC, Life HS (1990). The short Confusion Assessment Method (Short CAM): training manual and coding guide. Intern Med..

[39] Wei LA, Fearing MA, Sternberg EJ, Inouye SK (2008). The Confusion Assessment Method: a systematic review of current usage. J Am Geriatr Soc.

[40] Morandi A, McCurley J, Vasilevskis EE, Fick DM, Bellelli G, Lee P (2012). Tools to detect delirium superimposed on dementia: a systematic review. J Am Geriatr Soc.

[41] Boustani M, Campbell N, Munger S, Maidment I, Fox C. Impact of anticholinergics on the aging brain: a review and practical application. Aging Health. 2008;4(3).

[42] Egberts A, Moreno-Gonzalez R, Alan H, Ziere G, Mattace-Raso FUS. Anticholinergic drug burden and delirium: a systematic review. J Am Med Dir Assoc. 2020. 10.1016/j.jamda.2020.04.019 32703688

[43] McCusker J, Cole M, Abrahamowicz M, Primeau F, Belzile E (2002). Delirium predicts 12-month mortality. JAMA Intern Med..

[44] Rockwood K, Cosway S, Carver D, Jarrett P, Stadnyk K, Fisk J (1999). The risk of dementia and death after deliirum. Age Ageing.

[45] Knaus WA, Draper EA, Wagner DP, Zimmerman JE (1985). APACHE II: a severity of disease classification system. Crit Care Med..

[46] Charlson ME, Pompei P, Ales KL, MacKenzie CR (1987). A new method of classifying prognostic comorbidity in longitudinal studies: development and validation. J Chronic Dis..

[47] Waterlow J (1985). Pressure sores: a risk assessment card. Nurs Times.

[48] Sclan S, Reisberg B (1992). Functional assessment staging (FAST) in Alzheimer’s disease: reliability, validity and ordinality. Int Psychogeriatr IPA..

[49] Association AP, Association AP. Diagnostic and statistical manual of mental disorders (revised 4th ed.). Washington, DC: Author. 2000.

[50] Folstein MF, Folstein SE, McHugh PR (1975). “Mini-mental state”: a practical method for grading the cognitive state of patients for the clinician. J Psychiatr Res.

[51] StataCorp. Stata Statistical Software: Release 15. College Station, TX: StataCorp LLC; 2017.

[52] Han L, Allore HG, Araujo KL, Pisani MA (2016). Anticholinergic drug burden predicts delirium severity among older medical patients under intensive care. Alzheimer's Dement J Alzheimer's Assoc..

[53] Lertxundi U, Domingo-Echaburu S, Hernandez R, Peral J, Medrano J (2013). Expert-based drug lists to measure anticholinergic burden: similar names, different results. Psychogeriatrics..

[54] Davis DH, Barnes LE, Stephan BC, MacLullich AM, Meagher D, Copeland J (2014). The descriptive epidemiology of delirium symptoms in a large population-based cohort study: results from the Medical Research Council Cognitive Function and Ageing Study (MRC CFAS). BMC Geriatr..

[55] Salahudeen MS, Duffull SB, Nishtala PS (2015). Anticholinergic burden quantified by anticholinergic risk scales and adverse outcomes in older people: a systematic review. BMC Geriatr..

[56] Day HR, Perencevich EN, Harris AD, Gruber-Baldini AL, Himelhoch SS, Brown CH (2012). Association between contact precautions and delirium at a tertiary care center. Infect Control Hosp Epidemiol..

[57] Pan Y, Jiang Z, Yuan C, Wang L, Zhang J, Zhou J (2018). Influence of physical restraint on delirium of adult patients in ICU: a nested case-control study. J Clin Nurs..

[58] Thomas VS, Rockwood KJ (2001). Alcohol abuse, cognitive impairment, and mortality among older people. J Am Geriatr Soc..

[59] Vaurio LE, Sands LP, Wang Y, Mullen EA, Leung JM (2006). Postoperative delirium: the importance of pain and pain management. Anesth Analg..

